# Bioengineering of the Uterus

**DOI:** 10.1007/s43032-021-00503-8

**Published:** 2021-04-07

**Authors:** Yushi Yoshimasa, Tetsuo Maruyama

**Affiliations:** Department of Obstetrics and Gynecology, Keio University School of Medicine, 35 Shinanomachi, Shinjuku-ku, Tokyo, 160-8582 Japan

**Keywords:** Tissue engineering, Uterus, Endometrium, Stem cells, Scaffold

## Abstract

Impairment of uterine structure and function causes infertility, pregnancy loss, and perinatal complications in humans. Some types of uterine impairments such as Asherman’s syndrome, also known as uterine synechiae, can be treated medically and surgically in a standard clinical setting, but absolute defects of uterine function or structure cannot be cured by conventional approaches. To overcome such hurdles, partial or whole regeneration and reconstruction of the uterus have recently emerged as new therapeutic strategies. Transplantation of the whole uterus into patients with uterine agenesis results in the successful birth of children. However, it remains an experimental treatment with numerous difficulties such as the need for continuous and long-term use of immunosuppressive drugs until a live birth is achieved. Thus, the generation of the uterus by tissue engineering technologies has become an alternative but indispensable therapeutic strategy to treat patients without a functional or well-structured uterus. For the past 20 years, the bioengineering of the uterus has been studied intensively in animal models, providing the basis for clinical applications. A variety of templates and scaffolds made from natural biomaterials, synthetic materials, or decellularized matrices have been characterized to efficiently generate the uterus in a manner similar to the bioengineering of other organs and tissues. The goal of this review is to provide a comprehensive overview and perspectives of uterine bioengineering focusing on the type, preparation, and characteristics of the currently available scaffolds.

## Introduction

In 2014, transplantation of a uterus resulted in the successful birth of child in a patient with uterine agenesis [1]. That study represents the ultimate treatment for congenital and acquired uterine defects [2]. However, uterine transplantation has many obstacles, such as the shortage of donors, possible organ rejection, and the long-term use of immunosuppressive drugs [3].

**Fig. 1:**
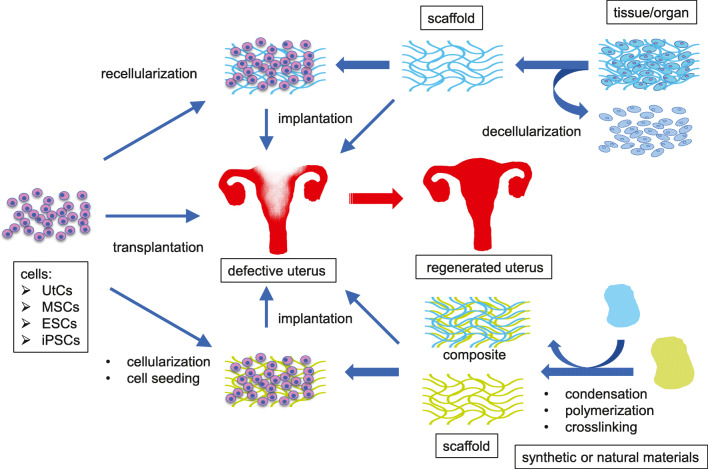
Therapeutic strategies for bioengineering of the uterus. Currently, scaffolds for the regeneration of the uterus are divided into two categories: (1) a decellularized scaffold that is prepared from the uterus or uterine tissue derived from the donor through removal of cellular components by single or combined physical, chemical, and enzymatic treatments; (2) synthetic or natural materials made of collagen, gelatin, fibrin, silk sponge, poly(glycolic acid), poly(glycerol sebacate), and poly(lactic-co-glycolic acid) through condensation, polymerization, and crosslinking. To regenerate the uterus, these scaffolds with or without the addition of various types of cells including uterine cells and mesenchymal stem cells are implanted directly into the defective uterus. Alternatively, the cells alone are transplanted directly into the defective uterus. UtCs, uterine cells; MSCs, mesenchymal stem cells; ESCs, embryonic stem cells; iPSCs, induced pluripotent stem cells

Bioengineering of a whole or partial uterus may overcome these limitations [3, 4]. In uterine tissue engineering, a uterus-like biomaterial is grafted into patients with uterine factor-associated reproductive and perinatal disorders, including infertility and recurrent pregnancy loss (Fig. 1). The material consists of either an acellular tissue-supporting material—termed a scaffold—alone or a scaffold repopulated with the patient’s own cells or those from an immunocompatible donor. The scaffold is necessary to support the repopulating cells structurally and functionally before or after grafting, although transplantation of cells or tissues such as organoids without support by the scaffold may have a potential for at least partial regeneration of the tissue. Because the acellular scaffold basically consists of extracellular matrices (ECM) alone, it exhibits no or very little immunogenicity even when it is derived from a mismatched unrelated donor. If an acellular scaffold were repopulated with the patient’s own cells, there would be no need for immunosuppressive drugs.

Based on the basic principles of organ tissue engineering, the following issues must be resolved at each step of uterine bioengineering. (1) What type of scaffold or template will be used? (2) How will the scaffold be prepared? (3) Will the scaffold be repopulated prior to grafting? (4) How will [re] cellularization be achieved? (5) What type of cells will be used for (re)-cellularization? (6) How will the uterus-like materials be grafted? [3, 4]. The gold standard of each step has yet to be established. In this review, we provide an overview and perspectives of bioengineering of the uterus, focusing on the type, preparation and characteristics of currently available scaffolds.

## General Aspects and Current Status of Organ Tissue Engineering

### Basic Strategy

The aim of organ tissue engineering (OTE) is to generate biological tissues and organs to treat a variety of medical conditions involving structural and functional impairment. The typical process of OTE consists of preparation of a cell/tissue-supporting material termed a scaffold, implantation of cells into the scaffold, repopulation, and remodeling of the scaffold by the cells, and thereafter grafting of the organ/tissue-like scaffold into a patient [5–7]. Alternatively, the scaffold can be grafted directly into the patient without repopulation and remodeling of the cells. The acellular scaffold supports the cells that migrate to it from the recipient’s body, allows the migrating cells to proliferate and differentiate, and eventually gives rise to the regenerated tissue and organ [5–7]. An example of the strategy used in a general OTE is shown in Fig. 1, focusing on the repair of a uterus.

### Cell Source

The typical strategy for OTE includes three processes: determining a proper cell source, processing the cells and choosing an appropriate supportive scaffold. As for the proper cell source, stem/progenitor cells are ideal because they have potential for generation of the desired types of tissues and organs through self-renewal and multilineage differentiation [8]. Adult stem cells (ASCs), embryonic stem cells (ESCs) and induced pluripotent stem cells (iPSCs) are the most likely candidates for the cell source for OTE [8].

#### Adult Stem Cells

Among the adult stem cells ( ASCs), mesenchymal stem cells (MSCs) show promise for a wide range of OTE and regenerative medicine applications [9]. MSCs can be isolated from numerous tissues, including bone marrow and adipose tissues. They can be cultured prior to clinical use [9]. Depending on the specific application, suspensions of MSCs collected from MSC-enriched tissue of the patient or an immune-compatible donor may then be introduced intravenously or by local injection to achieve the desired therapeutic effects, such as treating autoimmune diseases or stimulating local tissue repair and vascularization [9]. Indeed, MSCs achieve tissue repair without engraftment and differentiation but instead through paracrine signaling and communication through cell-cell contacts responsible for angiogenesis and immunomodulation [9].

MSCs may also be utilized for tissue engineering by first promoting their differentiation toward a desired cell type (e.g., osteoblasts, chondrocytes, and adipocytes) prior to surgical implantation, often along with scaffold material. Initial animal studies, however, revealed that MSC-derived chondrocytes do not show regenerative abilities, resulting in a failure of engraftment [10]. Thus, practical application of MSCs to OTE appears limited.

Besides MSCs, tissue-specific stem cells are also candidates for OTE. Tissue-specific stem cells produce differentiated cells that function as a part of their specific tissues and organs and also govern the maintenance of their tissue of origin. Thus, given the specified differentiation and regeneration potential of tissue-specific stem cells, it is reasonable to utilize them for OTE. However, there are several limitations in that (1) tissue-specific stem or progenitor cells have not been clearly identified in all types of tissues and organs, (2) they are often inaccessible and difficult to isolate and handle even if identified, and (3) they are difficult to expand in vitro and in vivo.

#### Embryonic Stem Cells

Embryonic stem cells (ESCs) are derived from the inner cell mass of blastocyst-stage embryos. They retain the ability to proliferate indefinitely in culture and retain their pluripotency, i.e., the capacity to differentiate into many cell types. Thus, the use of ESCs has long been considered an important therapeutic strategy for regenerative medicine, including OTE [11]. The establishment and availability of both mouse and human ESCs have facilitated this therapeutic strategy [11]. Indeed, approximately 30 clinical trials and numerous basic OTE studies using ESCs have been conducted or are ongoing [12]. ESC-based OTE, however, has limitations because of potential tumorigenic risks, the possibility of immune rejection, and ethical problems associated with the use of human embryos [6]. Furthermore, despite the pluripotency of ESCs, the efficiency of induction of differentiation into a desired cell type is less than 100% [6]. As a result of the inefficiency, tumors might arise from a small fraction of residual undifferentiated cells even after differentiation induction. These limitations have delayed clinical translation of ESC research [6].

#### Induced Pluripotent Stem Cells

To overcome the limitations of ESCs, particularly the risk of immune rejection and ethical problems associated with the use of human embryos, induced pluripotent stem cells (iPSCs) have emerged as a promising alternative cell source for regenerative medicine, including OTE. IPSCs can be generated from adult somatic cells and acquire ESC-like pluripotent stemness upon reprogramming through the forced expression of factors for maintenance of the defining ESC properties [13]. The reprogramming efficiency to generate iPSCs is, however, still not high [14]. Also, the differentiation efficiency of iPSCs into particular types of cells is not high, at least in part, because of heterogeneity in iPSCs and a lack of established protocols for induction of differentiation [15]. Furthermore, there is a possible risk of generating tumors in iPSC-based therapies [15]. Nevertheless, there is no requirement for human embryos and no or very little risks of immune rejections when using autologous or HLA-matched iPSCs, which has dramatically facilitated preclinical and clinical trials together with basic studies using iPSCs. Indeed, more than 70 clinical trials have been conducted or are ongoing [12]. The first clinical trial involved transplantation of a sheet of retinal pigment epithelial cells differentiated from autologous iPSCs in a patient with neovascular age-related macular degeneration [16]. Many observational or interventional studies involving ESCs and/or iPSCs have been registered in public databases. However, only a small part has focused on the actual transplantation of cells [12].

### Scaffolds

To efficiently achieve regeneration and reconstruction of organs and tissues, a supporting biomaterial(s) termed a scaffold is needed to endow a 3D structure that enables cell engraftment, tissue growth and differentiation. Ideally, the scaffolds should satisfy the following requirements: no adverse immunogenicity, good biocompatibility, no toxicity, timely biodegradability and appropriate biomechanical properties. Current scaffolds can be divided into 3 categories: natural materials, synthetic materials and natural acellular extracellular matrices after complete removal of the cells (decellularized matrices).

### Natural and Synthetic Materials

Natural biomaterials consist of pre-existing macromolecules that are present in ECM. They include collagen, gelatin, hyaluronic acid hydrogels, fibrin, glycosaminoglycans, alginate, Matrigel, silk, hydroxyapatite, and others [17]. These materials exhibit specific advantages, including mechanical and adhesive properties similar to natural ECM. In addition to good biocompatibility, they show less immune responsiveness and possess little capacity for initiating signals. These materials have some shortcomings, including batch variability, a short degradation period, difficulty in purification, and quality control.

To overcome the obstacles associated with natural biomaterials, synthetic scaffolds have been developed and now can be divided into 4 types: polymers, ceramics, metals and graphene [17]. Although there are differences in properties among these materials, the general advantages of the synthetic scaffolds include easy modification, designable properties and good mechanical strength. Conversely, they are characterized by poor cell adhesion properties, poor biological signals and poor biocompatibility. No or poor bioresorbability of synthetic materials can be either beneficial or detrimental for OTE.

Among the synthetic materials, polymers are the most prevalent type including polylactic acid (PLA), poly (lactic-co-glycolic acid) (PLGA), polycaprolactone (PCL), polyethylene glycol (PEG), polyhydroxyl ethyl methacrylate (PHEMA), and polyvinyl alcohol (PVA) [17]. Lactic acid polymers were invented in the eighteenth century and are now widely used in a variety of fields. PLA and PLGA are superior to the other synthetic polymers in terms of biocompatibility, biodegradability, bioresorbability, low immunogenicity and low toxicity. Thus, PLA and PLGA are favorably applied as 3D scaffolds in various medical fields, including dentistry and plastic surgery. In addition to the simple use of one synthetic material, combinatory use of synthetic materials together with or without bioactive substances improves the scaffolds’ properties resulting in successful OTE through facilitation of cell fabrication, proliferation and differentiation [5]. For instance, PCL was mixed with PLA to improve the thermal resistance and mechanical properties of engineered tissues [18].

As an alternate to a scaffold-based OTE, cell sheet tissue engineering is a scaffold-free strategy for creating transplantable two-dimensional (2D) and three-dimensional (3D) tissues and organs [19]. Cell sheet technology consists mainly of a “thermo-responsive culture dish” that is coated with poly(N-isopropylacrylamide) (PIPAAm). This material changes from a hydrophilic state to a hydrophobic state when the temperature is dropped from 37°C to 32°C. This culture dish enables reversible cell adhesion and detachment by thermo-controllable hydrophobicity of the surface. This material permits non-destructive harvest of cultured cells as an intact monolayer cell sheet, including the deposited ECM. Layering of these cell sheets enables the fabrication of a 3D tissue. Cell sheet-based tissues and their transplantation are used in many settings, such as the heart, cornea, esophagus, periodontal procedures, the middle chamber of the ear, knee cartilage and lung [19].

#### Decellularized Matrices

Decellularization is defined as a multi-step process of removing the viable cellular components from a human or animal organ or tissue to create a scaffold with retained macrostructure and microstructure of the ECM components, including collagen, elastin, microfibrils, proteoglycans, glycosaminoglycans (GAGs) and various growth factors [20].

Decellularizing procedures involve a blend of chemical, physical, and enzymatic treatments and vary depending on the origin and property of the tissue being processed [21]. Chemical treatments include acids and bases, hypotonic and hypertonic solutions, detergents such as Triton X-100, Triton X-200, sodium dodecyl sulfate (SDS), sodium deoxycholate (SDC), sulfobetaine-10 and -16, and solvents such as alcohols, acetone, ethylenediaminetetraacetic acid and tributyl phosphate. Physical methods include freeze-thaw cycles, direct application of force and pressure, and electroporation. Enzyme treatments include nucleases, trypsin, and Dispase. The reagents and methods used for decellularization may damage the microstructure and composition of the resultant scaffold and therefore may affect the biological and mechanical properties of the final product [21]. Thus, the choice of the reagents together with the methods is critically important.

In addition to the use of ECM derived from decellularized tissue, 3D ECM scaffolds prepared by whole organ decellularization have been explored in regenerative medicine and tissue engineering strategies [21]. ECM-based clinical products are prepared from various allogeneic or xenogeneic tissue sources, including dermis, urinary bladder, small intestine, mesothelium, pericardium, and heart valves, and from several different species, some of which are commercially available [21].

## Uterine Tissue Engineering

This review focuses on studies that aim to develop uterine tissue engineering with and without the use of exogenous cells. Various types of engineered 3D uterine tissue culturing systems have been developed and employed to study the mechanisms underlying endometrial differentiation and embryo implantation [3]. We also address the development of tissue culturing system.

### Cell source for Bioengineering of the Uterus

When employing exogenous cells for the OTE of the uterus, ASCs including MSCs, ESCs, and iPSCs are the most likely candidates for repopulating the structure.

#### Uterus-Specific Stem Cells

Various types of uterine stem/progenitor cells have been isolated and identified [22, 23]. The main components of the uterus are the endometrium and myometrium. Thus, those tissues have been used as sources of stem/progenitor cells [24, 25].

Several types of transplantable, i.e., prospectively isolatable endometrial stem/progenitor cells have been identified, including CD140b^+^CD146^+^ or SUSD2^+^ endometrial mesenchymal stem cells (eMSCs), N-cadherin^+^ endometrial epithelial progenitor cells and side population (SP) cells, a heterogeneous population predominantly comprised of endothelial cells [23]. In particular, SP cells have several stem/progenitor cell properties. Unfortunately, they are present at low frequencies in the original tissue and organ and therefore, it is extremely difficult to obtain a sufficient number of SP cells for OTE [26, 27]. Furthermore, endometrial SP cells require appropriate an microenvironment and supporting cells, i.e., a niche, to maximally support stem/progenitor cell activities, including cell differentiation [28]. Indeed, in vivo endometrial tissue reconstitution activity is low when SP cells alone are transplanted into immunodeficient mice [27]. However, the activity increases when they are co-transplanted with whole endometrial cells [28].

Like endometrial stem/progenitor cells, several types of myometrial stem/progenitor cells have been identified: SP cells [29], CD34+/CD49f+ cells [30], CD44+/Stro-1+ cells [31] and CD140b+/CD146+ or SUSD2+ cells [32]. Although the percentage of these stem cells varies, only 3% of whole myometrial cells are myometrial SP cells or CD34+/CD49f+ cells [29, 30]. Furthermore, the myometrial tissue reconstitution ability of these stem cells alone is low [29, 30].

Thus, although endometrial and myometrial stem/progenitor cells are attractive and promising candidate cell sources for bioengineering of the uterus, there remain several problems, including the difficulty of in vitro and in vivo expansion, that make it difficult to use them for clinical applications.

#### ESCs and iPSCs for Bioengineering of the Uterus

As previously mentioned, ESCs and iPSCs can proliferate indefinitely, maintaining their stemness. Therefore, the use of these cells could theoretically overcome the difficulties described above. If a proper method of differentiation of ESCs and iPSCs into each component of the uterus were developed, the use of ESCs and iPSCs would be valuable for the bioengineering of the uterus. Human ESCs have the potential for generating endometrial cells both in vitro and vivo [33, 34]. Furthermore, Miyazaki et al. successfully directed the differentiation of human iPSCs through intermediate mesoderm, coelomic epithelium, and Müllerian duct to endometrial stromal fibroblasts under molecularly defined embryoid body culture conditions using specific hormonal treatments [35].

### Scaffolds for the Bioengineering of the Uterus

Similar to the bioengineering of other organs, natural, synthetic or decellularized ECM materials have been used in both basic and clinical studies of the bioengineering of the uterus (in Tables 1 and 2).

**Table 1:** Studies of uterine tissue engineering using synthetic materials, natural materials or cell sheets

Target species	Target tissue	Size of graft	Scaffold material	Cells used	Cell culture time in vitro	Histological tests in vivo	Pregnancy test	References
Human	Myometrium	1 × 1.5 cm	Polyglactin-910 (Vicryl) mesh scaffold	Human myometrial cells	3 weeks	N/A	N/A	Young et al., 2003 [36]
Rabbit	Full thickness	1 mL/well (12-well plate)	Collagen/Matrigel	Rabbit uterine cells as filled cells and mouse embryo	14 days	N/A	N/A	Lu et al., 2009 [37]
Rabbit	Endometrium	4, 12, 96-well plates	Collagen	Rabbit endometrial stromal and epithelial cells	14 days	N/A	N/A	Wang et al., 2010 [38]
Rat	Full thickness	1.5 (length) × 0.5 (width) × 0.1 (thickness) cm	Collagen	No (only basic fibroblast growth factor [bFGF])	N/A	90 days	90 days post transplantation	Li et al., 2011 [39]
Rat	Full thickness	-	Collagen	No (vascular endothelial growth factor [VEGF])	N/A	90 days	90 days post transplantation	Lin et al., 2012 [40]
Rat	Full thickness	1.5 (length) × 0.5 (width) × 0.1 (thickness) cm	Collagen	Rat bone marrow–derived MSCs	3 days	90 days	90 days post transplantation	Ding et al., 2014 [41]
Rat	Full thickness	1.5 (length) × 0.5 (width) x ~0.04 (thickness) cm	Collagen	Endometrium-like cells differentiated from human ESCs	N/A	12 weeks	12 weeks post transplantation	Song et al., 2015 [34]
Human	Endometrium	6 (diameter, circular shape) × 3 (thickness) mm	Collagen	Human endometrial carcinoma cell line (Ishikawa) and human umbilical vein endothelial cell line	14 days	N/A	N/A	Pence et al., 2015 [42]
Rat	Full thickness	Injected fibers	Collagen	Human umbilical cord–derived MSCs	N/A	60 days	60 days post transplantation	Xu et al., 2017 [43]
Human	Endometrium	4 × 6 cm	Collagen	Human autologous bone marrow mononuclear cells	24 hours	3 menstrual cycles	5/5 patients gave birth	Zhao et al., 2017 [44]
Human	Endometrium	4 × 6 cm	Collagen	Human autologous umbilical cord–derived MSCs	N/A	3 months	10/26 patients became pregnant	Cao et al., 2018 [45]
Rat	Full thickness	1.5 (length) × 0.5 (width) cm	Collagen	No (leukemia inhibitory factor [LIF])	N/A	12 weeks	8 weeks post transplantation	Xue et al., 2019 [46]
Rat	Endometrium	2.5 × 0.5 cm	Collagen	Human umbilical cord–derived MSCs	3 days	60 days	60 days post transplantation	Xin et al., 2019 [47]
Rat	Full thickness	1.5 × 0.5 cm	Collagen	Human endometrial perivascular cells	N/A	90 days	90 days post transplantation	Li et al., 2019 [48]
Human	Endometrium	8 (punch biopsy) × 0.75 (thickness) mm	Collagen	Human stromal cells and endometrial organoids	10 days	N/A	N/A	Abbas et al., 2020 [49]
Human	Cervix	10 × 35 × 1 (thickness) mm	Silk sponge	Human cervical cells	8 weeks	N/A	N/A	House et al., 2010 [50]
Human	Cervix	8 (diameter, circular shape) × 4 (thickness) mm	Silk sponge	Human cervical cells	12 weeks	N/A	N/A	House et al., 2012 [51]
Human	Cervix	6 (diameter, circular shape) × 4 (thickness) mm	Silk sponge	Human cervical cells	4 weeks	N/A	N/A	House et al., 2014 [52]
Human	Cervix	8 (diameter, circular shape) × 6 (thickness) mm	Silk sponge	Human cervical cells	4 weeks	N/A	N/A	House et al., 2018 [53]
Human	Stromal cells	24-well plate	Hydrogel	Human endometrial stromal cells	7 days	N/A	N/A	Li et al., 2011 [54]
Human	Endometrium	4, 6, 12-well plates	Fibrin-agarose	1. Human endometrial epithelial and stromal cells 2. Human endometrial adenocarcinoma cell line and immortalized human endometrial stromal cell line	7 days	N/A	N/A	Wang et al., 2012 [55]
Human	Endometrium	4, 12-well plates	Fibrin-agarose	Human endometrial adenocarcinoma cell line and immortalized human endometrial stromal cell line	10 days	N/A	N/A	Wang et al., 2013 [56]
Human		24, 96-well plates	Gelatin	Human endometrial stem cells	28 days	N/A	N/A	Azami et al., 2013 [57]
Human		-	Collagen/carbon nanotubes composite	Human decidua parietalis stem cells	6 days	N/A	N/A	Sridharan et al., 2013 [58]
Human		1.5 × 1.5 cm	Gelatin/polyamide	Human endometrial MSCs	28 days	N/A	N/A	Su et al., 2014 [59]
Human		2.5 × 1 cm	Gelatin/polyamide	Human endometrial MSCs	N/A	90 days	N/A	Edwards et al., 2015 [60]
Human	Cervix	12-well plate	Polystyrene	Human endocervical cells (stroma + mucosal epithelium)	28 days	N/A	N/A	Arslan et al., 2015 [61]
Bovine	Endometrium	13 mm (diameter)	Electrospun polyglycolic acid (PGA)	Cattle endometrial stromal and epithelial cells	14 days	N/A	N/A	MacKintosh et al., 2015 [62]
Human	Cervix	5 (diameter, circular shape) × 1 (thickness) mm	Free	Human cervical cells	10 days	N/A	N/A	Gregorio et al., 2017 [63]
Human	Endometrium	12-well plate	Collagen/Matrigel	Human endometrial CD146+ cells	10 days	N/A	N/A	Fayazi et al., 2017 [64]
Rat	Endometrium	96-well plate	Heparin-poloxamer	Mouse endometrial epithelial cells (in vitro test)	3 days	14 days	N/A	Zhang et al., 2017 [65]
Rat	Endometrium	6-well plate	Heparin-modified poloxamer	Mouse endometrial epithelial cells (in vitro test)	4 hours	7 days	90 days post transplantation	Xu et al., 2017 [66]
Rat	Endometrium	6, 96-well plates	Heparin-modified poloxamer/ε-polylysine	Human endometrial carcinoma cell line (in vitro test)	4 hours	3 days	N/A	Xu et al., 2017 [67]
Rat	Endometrium	24-well plate	Pluronic F-127	Rat bone marrow stromal cells	7 days	2 weeks	N/A	Yang et al., 2017 [68]
Human	Endometrium	15 (diameter, circular shape) × 0.4 (thickness) mm	Polymerizable high internal phase emulsion	1. Human endometrial epithelial and stromal cells 2. Human endometrial adenocarcinoma cell line	15 days	N/A	N/A	Eissa et al., 2018 [69]
Human	Endometrium	10 (diameter, circular shape) × 0.2 (thickness) mm	Polymerizable high internal phase emulsion/fibronectin	Human endometrial stomal cells	9 days	N/A	N/A	Richardson et al., 2018 [70]
Rat	Endometrium	15 (length, capillary tube) × 1.2 (inner diameter) mm (in vivo test)	Gelatin methacryloyl/alginate	No (in vivo test), HepG2 (in vivo test)	10 days	6 weeks	N/A	Cai Y et al., 2018 [71]
Rat	Endometrium	1.5 (length) × 0.5 (width) × 0.1 (thickness) cm	Poly(glycerol sebacate) (PGS), Poly(lactic-co-glycolic acid) (PLGA), Collagen	Rat bone marrow–derived MSCs	N/A	90 days	90 days post transplantation	Xiao et al., 2019 [72]
Rat	Full thickness	2 × 1 cm	Silk fibroin-bacterial cellulose	Human endometrial cells, rat uterine cells	7 days	90 days	90 days post transplantation	Cai H et al., 2019 [73]
Mouse	Endometrium	96-well plate	Hyaluronic acid (HA) Hydrogel/fibrinogen/thrombin	Mouse endometrial stromal cells	24 hours	14 days	14 days post transplantation	Kim et al., 2019 [74]
Rat	Endometrium	2, 24, 96-well plates	HA hydrogel	Human bone marrow–derived MSCs	3 days	7 days	7 days post transplantation	Liu et al., 2019 [75]
Rabbit	Full thickness	6–8 (length) × 2.5 (width) × 0.2 (thickness) cm	PGA/PLGA	Rabbit endometrial and myometrial cells	N/A	6 months	6 months post transplantation	Magalhaes et al., 2020 [76]
Rat	Full thickness	0.5 (diameter, tubular shape) × 2.5 (length) cm	Boiled blood clots molded into tubular shapes	No	N/A	12 weeks	4, 8, 12 weeks post transplantation	Campbell et al., 2008 [77]
Rat	Endometrium	Circumferentially full length of the uterus from the cervix to the fallopian tube	Cell sheet	Rat oral mucosal epithelial cells	N/A	8 days	N/A	Kuramoto, et al., 2015 [78]
Rat	Endometrium	Circumferentially 10 mm (length)	Cell sheet	Rat endometrial cells	N/A	4 weeks	6 weeks post transplantation	Kuramoto, et al., 2018 [79]
Rat	Endometrium	1.5 (length) × 0.5 (width) cm	Cell sheet	Rat adipose-derived stem cells	N/A	60 days	60 days post transplantation	Sun et al., 2018 [80]

**Table 2:** Studies of uterine tissue engineering using decellularized scaffolds

Scaffold material	Scaffold size	Decellularized method	Decellularized reagent	Recellularization cells	Recellularization method	Cell culture time in vitro	Histological tests in vivo	Target species	Target tissue	Target size	Graft size	Pregnancy test	References
Human myometrium Rat myometrium	2 × 2 × 10 mm (human), 15 × 20 mm (rat)	Immersion with shaking	Ethanol and trypsin	Human and rat myocytes	Cultured on scaffold with shaking	51 days	N/A	N/A	N/A	N/A	N/A	N/A	Young et al., 2013 [81]
Rat full thickness uterus	Whole uterus	Perfusion via the aorta	SDS	Rat neonatal uterine cells + rat adult uterine cells + rat MSCs	Injected to whole uterine wall	10 days	90 days	Rat	Full thickness	1.5 cm (length) × 1/2 of the total circumference	1.5 × 0.5 cm	28 days	Miyazaki et al., 2014 [82]
Rat full thickness uterus	Whole uterus	Perfusion via the aorta	SDS	N/A	N/A	N/A	8 weeks	Rat	Full thickness	1.5 cm (length) × 1/2 of the total circumference	1.5 × 0.5 cm	8 weeks	Miki et al., 2019 [83]
Rat small intestine	15 mm (length) (intestine)	Immersion with shaking	SDS	N/A	N/A	N/A	8 weeks	Rat	Full thickness	1.5 cm (length) × 1/2 of the total circumference	1.5 × 0.5 cm	8 weeks	Miki et al., 2019 [83]
Rat full thickness uterus	15 × 5 mm	Immersion	SDS, Triton X-100	N/A	N/A	N/A	30 days	Rat	Full thickness	15 × 5 mm	15 × 5 mm	30 days	Santoso et al., 2014 [84]
Rat full thickness uterus	15 × 5 mm	High hydrostatic pressure	Saline solution only	N/A	N/A	N/A	30 days	Rat	Full thickness	15 × 5 mm	15 × 5 mm	30 days	Santoso et al., 2014 [84]
Mouse full thickness uterus	10 × 2 mm, 5 × 2 mm	Immersion	SDS	N/A	N/A	N/A	28 days	Mouse	Full thickness	5 × 2 mm	5 × 2,10 × 2 mm (pregnancy test)	30 days	Hiraoka et al., 2016 [85]
Rat full thickness uterus	Whole uterus	Perfusion via the aorta, only perfusion	DMSO + triton X-100, SDC	N/A	N/A	N/A	N/A	N/A	N/A	N/A	N/A	N/A	Hellström et al., 2014 [86]
Rat full thickness uterus	Whole uterus	Perfusion via the aorta + freeze-thaw	DMSO + triton X-100	N/A	N/A	N/A	N/A	N/A	N/A	N/A	N/A	N/A	Hellström et al., 2014 [86]
Rat full thickness uterus	Whole uterus	Perfusion via the aorta	DMSO + triton X-100, SDC	Rat endometrial and myometrial cells + rat MSCs	Injected to 20 × 5 mm patch	3 days	3 months	Rat	Full thickness	10 × 5 mm	10 × 5 mm	6 weeks	Hellström et al., 2016 [87]
Sheep full thickness uterus	Whole uterus	Perfusion via the uterine artery	SDS + DNase, SDC + DNase, SDC + Triton X-100 + DNase	Sheep fetal bone marrow stem cells	Injected to the ring shape scaffold	14 days (0.3–0.5 mm in thickness, ring shape)	N/A	N/A	N/A	N/A	N/A	N/A	Tiemann et al., 2020 [88]
Human amniotic membrane + poly (ester urethane)	N/A	Immersion	Triton X-100 + DNase Hypertonic saline + DNase, Lipase + DNase, Triton X-100 + lipase + DNase	Rabbit esophageal smooth muscle cells (in vitro only)	Cultured on the scaffold	10 days (6.4 mm in diameter, punched)	10 months	Rabbit	Uterus (thickness is unknown)	N/A	1 × 1 cm	N/A	Shi et al., 2015 [89]
Porcine full thickness uterus	Whole uterus	Perfusion via the uterine artery ± freeze-thaw	SDS	Human endometrial stromal and epithelial side population cells	Cultured on the scaffold	12 days (5 mm in diameter, punched)	N/A	N/A	N/A	N/A	N/A	N/A	Campo et al., 2017 [93]
Rabbit uterus	Whole uterus	Perfusion via the uterine artery	SDC + Triton X-100 + DNase	Rabbit embryo (as an implantation model)	Cultured on the hydrogel derived from the powder of the decellularized endometrium	48 hours	N/A	N/A	N/A	N/A	N/A	N/A	Campo et al., 2019 [94]
Human endometrium	~1–2 cm^2^ × 0.5 mm	Immersion with shaking	SDS + Triton X-100 + ribonuclease + DNase	Human endometrial cells	Cultured on the scaffold with insert	28 days (8 mm in diameter, punched × 0.5 mm in thickness)	N/A	N/A	N/A	N/A	N/A	N/A	Olalekan et al., 2017 [95]
Human amniotic membrane	N/A	Immersion with stirring	EDTA	Rat oral mucosal epithelial cells	Cultured on the scaffold	N/A	28 days	Rat	Endometrium	Scraped (N/A)	N/A	N/A	Chen et al., 2018 [90]
Human amniotic membrane	N/A	Immersion with stirring	EDTA	N/A	N/A	N/A	28 days	Rat	Endometrium	Scraped (N/A)	N/A	N/A	Chen et al., 2019 [91]
Human amniotic membrane	2.5 × 2.5 cm	Immersion	EDTA	Rat oral mucosal epithelial cells	Cultured on the scaffold	10 days	28 days	Rat	Endometrium	Scraped (N/A)	N/A	28 days	Chen et al., 2019 [92]
Sheep full thickness uterus	Whole uterus	Perfusion via the uterine artery perfusion + shaking	SDS + Triton X-100, DMSO + Triton X-100 (perfusion only), SDS (perfusion + shaking)	N/A	N/A	N/A	10 days	Rat	Full thickness	10 mm longitudinal incision only	10 × 5 × 5 mm	N/A	Daryabari et al., 2019 [96]
Rabbit uterus	Whole uterus	Immersion with shaking	SDS + Triton X-100	Human umbilical vein endothelial cells	Cultured on the scaffold (1 × 1 cm)	48 hours	90 days	Rat	Subcutaneous and uterus full thickness	1 cm (length)	1 cm (length)	N/A	Yao et al., 2020 [97]
Rat uterus	Segment cut into	Immersion with shaking	SDS + Triton X-100	N/A	N/A	N/A	7 days	Rat	Endometrium	N/A	50 μL of the aloe-poloxamer hydrogel with ECM nanoparticles and E2	N/A	Yao et al., 2020 [98]

#### Synthetic Materials, Natural Materials, or Cell Sheet-Based Strategy

Since the early 2000’s, synthetic materials natural materials and cell sheets have been explored for bioengineering of the uterus (Table 1) [34, 36–80]. Most studies have used collagen-based or collagen-containing natural materials [34, 37–49, 58, 64]. Target species have included humans [36, 42, 44, 45, 49–61, 63, 64, 69, 70], rats [34, 37–41, 43, 46, 48, 65–68, 71, 73, 75, 77–80] and others. The target tissue of most studies is the endometrium [38, 42, 44, 45, 47, 49, 55, 56, 62, 64–72, 74, 75, 78–80]. Stem cells, including MSCs and ESCs, have been used for in vitro culture, repopulation of scaffolds and/or in vivo transplantation [34, 41, 43, 45, 47, 59, 60, 72]. Two clinical trials have been conducted to explore the regeneration of endometrium and pregnancy using tissue engineering technologies [44, 45]. Zao et al. used a collagen scaffold to treat human patients with severe Asherman’s syndrome [44]. They aspirated the patients’ bone marrow and mononuclear cells (BMNCs) were isolated. Five patients with Asherman’s syndrome received a uterine transplant of a collagen scaffold seeded with autologous BMNCs. Over three menstrual cycles post-surgery, hysteroscopy and biopsy were performed to evaluate the endometrial status, and all of the patients achieved pregnancy and gave birth to a living child. Moreover, implantation of the BMNC-collagen scaffold onto the uterine lining downregulated ΔNp63 expression, reversed the associated pathological changes, normalized the stemness alterations and restored endometrial regeneration. Cao et al. proved the validity of allogenic cell therapy for recurrent intrauterine adhesion (IUA) patients using umbilical cord-derived mesenchymal stromal cells (UC-MSCs) loaded onto a collagen scaffold [45]. Twenty-six patients were enrolled in this clinical trial and 10 out of the patients achieved pregnancy, leading to 8 live births with no obvious birth defects and no placental complications. Spontaneous abortions were observed in 1 patient in the third trimester of pregnancy and another at 7 weeks.

Thus, there have been numerous basic and clinical studies exploring the use of synthetic materials and natural materials. Future analyses should determine which of the materials is optimal for bioengineering of the uterus. Critical parameters include in vivo characteristics rather than those in vitro, properties of the biomaterials and support of pregnancy. Furthermore, the use of larger animals, ideally primates, would enhance the characterization of the materials and methods used. In this context, the study conducted by Magalhaes et al. may provide useful information. They used a polyglycolic acid (PGA)/PLGA scaffold seeded with autologous cells to restore uterine structure and function in rabbits [76]. Rabbits underwent a subtotal uterine excision and were reconstructed with a scaffold seeded with autologous endometrial and myometrial cells. At 6 months post-implantation, the cell-seeded engineered uteri developed native tissue-like structures, including organized luminal/glandular epithelium, stroma, vascularized mucosa, and a two-layered myometrium. The rabbits had normal pregnancies (4 in 10) in the reconstructed segment of the uterus and supported fetal development to term and live birth.

As a unique alternative to a scaffold-based OTE, cell sheet tissue engineering has been used for uterine endometrial repair [78–80]. In 2015, Kuramoto et al. showed that the transplantation of oral mucosal epithelial cell sheets prevented IUA in rats [78]. Moreover, the same group reported in 2018 that rat endometrial cell sheets could repair IUA leading to successful pregnancies in the regenerated endometrium [79]. In 2018, Sun et al. showed that cell sheet engineering using adipose-derived stem cells (ADSCs) repaired IUA in rats and that pregnancy could be achieved 60 days after transplantation [80]. They also found that ADSCs were mainly detected in the basal layer of the regenerating endometrium and that some ADSCs differentiated into endometrial stromal-like cells and muscle cells and also stimulated angiogenesis. Given the encouraging results obtained in the 3 studies, cell sheet therapy for OTE is being explored in clinical settings [19]. Cell sheet technologies are promising as a new therapeutic strategy for endometrial damage. However, those technologies still have limitations. For example, it is difficult to achieve multilayered cell sheets in vitro. Moreover, large scale production of differentiated cells with vascularized thick tissues is difficult [19]. Thus, there are considerable obstacles to be overcome in the regeneration and reconstruction of large portions of the uterus.

#### Decellularization and Recellularization Strategy

Decellularization and recellularization techniques for regeneration of the uterus have emerged since 2013 as shown in Table 2 [81–98].

In 2013, Young et al. were the first to use a decellularized matrix prepared from rat and human myometrium for in vitro uterine tissue engineering in 2013 [81]. Miyazaki and Maruyama demonstrated for the first time that the decellularized scaffold prepared from rat uterus had the potential for use as a supportive material to regenerate functional uterine tissue both in vitro and in vivo [82]. An acellular ECM scaffold together with a perfusable vascular architecture was prepared from rat uteri through decellularization by aortic perfusion with detergents such as SDS. Uterine-like tissues were then regenerated and maintained in vitro for up to 10 days through in vitro recellularization of the scaffold with adult and neonatal rat uterine cells and rat MSCs followed by aortic perfusion in a bioreactor. Moreover, placement of an acellular scaffold onto a partially excised rat uterus promoted recellularization and regeneration of uterine tissues and achievement of pregnancy nearly comparable to that in an intact uterus [82]. The same group showed that disoriented placement of the scaffold onto a partially excised rat uterus resulted in regeneration of the uterine tissue but with aberrant structures including ectopic location of glands and an abnormal lining of smooth muscle layers [83]. They also prepared an ECM scaffold from rat small intestine, but, unlike the uterine scaffold, it had no supportive capacity. These results collectively indicate that the ECM and architecture of the uterine scaffold retain functionality and determine the orientation and topology of regenerated uterine tissue [83]. Santoso et al. [84] and Hellström et al. [86, 87] independently demonstrated that uterine scaffolds prepared by different protocols had similar capacities as supportive materials to regenerate uterine tissue in rats. To prepare the decellularized uterine scaffold, Santoso et al. employed SDS or high hydrostatic pressure [84]. Hellström et al. used 3 different protocols—DMSO plus Triton-X100 followed by washing with PBS or distilled water, or SDS. They found that DMSO plus Triton-X100-generated scaffolds were preferable [87]. Tiemann et al., in the same group headed by Hellström, showed that perfusion with SDC is a favorable treatment for preparation of decellularized sheep uterine scaffold capable of supporting stem cells for 2 weeks in vitro [88].

Several groups prepared decellularized uterus-related or unrelated scaffolds from humans or from animals larger than rodents and used them for in vitro or in vivo uterine tissue engineering. Shi et al. and Chen et al. used human amniotic membrane as a xenograft and ectopic scaffold to repair the injured endometrium of rats or rabbits [89–92]. Campo et al. prepared decellularized porcine uterine scaffolds and recellularized them with only human endometrial stromal and epithelial SP cells (stem-like cells) for in vitro study [93]. They also decellularized whole rabbit uterus by a perfusion procedure via the uterine artery, followed by microdissection, lyophilization, milling, partial digestion and freezing [94]. A rabbit embryo was cultured in vitro on a hydrogel derived from powdered decellularized endometrium as an implantation model [94]. Olalekan et al. prepared decellularized human endometrial tissue for a novel 3D endometrium in vitro model [95]. It was repopulated with primary endometrial cells. Daryabari et al. found that perfusion with SDS and preservation in formalin could be used for preparation of a decellularized ovine uterine scaffold that was capable of regenerating the uterus when grafted into the uteri of rats [96]. Yao et al. decellularized whole rabbit uteri for xenografting to rat full thickness uterine walls [97]. They also decellularized a segment of rat uterus by an immersion procedure, pulverized it into a powder, and mixed it with aloe-poloxamer hydrogel and estradiol [98]. They injected the hydrogel into the injured uterine wall to prevent IUA in a rat model.

Overall, most studies have employed chemical treatments using ionic detergents such as SDS to isolate decellularized uterine scaffolds (Table 2). However, Padma et al. pointed out that non-ionic detergents such as Trion X-100 were milder than ionic detergents and therefore minimized the denaturing of ECM proteins [99]. Thus, it remains to be determined which protocol and which biomaterial should be employed for the bioengineering of the uterus. Like synthetic and natural materials, in vivo characteristics of decellularized scaffolds rather than those in vitro are critical in the choice of protocol and biomaterial. Furthermore, the use of animals larger than rats and mice, ideally primates, would be better for the characterization and validation of a decellularized scaffold in terms of clinical applications. On the other hand, compared to synthetic or natural materials, the ECM and architecture preserved in a decellularized scaffold may determine the orientation and topology of the regenerated uterine tissue [83]. Therefore, in clinical testing of bioengineering methods, it would be preferable if the decellularized scaffold were prepared from a human uterus followed by transplantation in a proper orientation to fabricate the complex structure of the uterus.

## Perspectives

Bioengineering studies of the uterus have relied upon a variety of scaffolds materials, including natural, synthetic and decellularized ECM. These studies are promising, suggesting clinical approaches to the repair of defective uteri. Nevertheless, several obstacles remain. One of them is the difficulty of in vitro repopulation of the (whole) uterine scaffold, a process that is absolutely required for regeneration of a whole uterus. As mentioned previously, many types of stem/progenitor cells, including endometrial SP cells, need an appropriate microenvironment (a niche) to exhibit maximal stem cell functions such as self-renewal, expansion and production of daughter cells that differentiate into one or multiple lineages [100, 101]. Thus, the full repopulation of the scaffold and maintenance of the resultant regenerated uterus requires a large and sufficient number of mature and/or differentiated uterine cells capable of supporting stem/progenitor cells. To obtain a sufficient amount of such cells, iPSCs and ESCs, especially the former, are needed as a cell source. A proper method of differentiation of ESCs and iPSCs into uterine cells, however, is largely unknown, although a few studies have addressed this issue [33–35].

In addition to selecting a cell source and a differentiation-inducing protocol, efficient methods of in vitro repopulation of the uterine scaffolds remains to be established. Several studies have reported successful repopulation of uterine scaffolds. However, their sizes have been relatively small (Tables 1 and 2). A few studies have attempted to repopulate whole rat uterine decellularized scaffolds through direct cell injection and/or perfusion, but the repopulation required a huge number of cells, including stem/progenitor cells [82, 87, 88]. It appears that the repopulation efficiency is low. Furthermore, the repopulated scaffolds were difficult to maintain in vitro for a long period [82, 87, 88]. Although repopulation with iPSCs has been accomplished up to human scale for several other organs including the heart, there remain limitations in obtaining a sufficient number of different types of cells for repopulation [102]. Given that repopulation depends on perfusion and/or injection, the precise spatial positioning of different types of repopulating cells is challenging to achieve [102]. Recently, a 3D bioprinting technique has been developed as a manufacturing process [102]. In this approach, biocompatible materials such as cells and growth factors are used as “inks” to print living tissue-like structures layer-by-layer. This approach has emerged as a new strategy for fabrication of complex biological constructs in the field of tissue engineering and regenerative medicine. Bioprinting has the potential to overcome some of the repopulation-related limitations and to substantiate the merit of the scaffold-based uterine tissue engineering.

## Conclusions

We here provide an overview and perspectives of uterus bioengineering, emphasizing the type, preparation and characteristics of the currently available scaffolds. There remain many obstacles rendering bioengineering of the whole uterus quite difficult. However, partial regeneration of the uterus through scaffold-based uterine tissue engineering is feasible because bare uterine scaffolds have the potential to at least partially regenerate the uterus through their support for the migration, proliferation and differentiation of primitive cells present in the neighboring uterine tissues. Initially, the bioengineering of the uterus will be clinically applied to treatment of partial defects of the endometrium due to Asherman’s syndrome, partial or whole defects of the cervix due to conization and trachelectomy and partial defects of the myometrium due to segmental resection of the uterus.

## Data Availability

Not applicable.
